# Speech perception is similar for musicians and non-musicians across a wide range of conditions

**DOI:** 10.1038/s41598-019-46728-1

**Published:** 2019-07-18

**Authors:** Sara M. K. Madsen, Marton Marschall, Torsten Dau, Andrew J. Oxenham

**Affiliations:** 1 Hearing Systems Group, Department of Health Technology, Technical University of Denmark, Ørsteds Plads, 2800 Lyngby, Denmark; 2 Department of Psychology, University of Minnesota, 75 East River Parkway, Minneapolis, MN 55455 USA

**Keywords:** Auditory system, Human behaviour

## Abstract

It remains unclear whether musical training is associated with improved speech understanding in a noisy environment, with different studies reaching differing conclusions. Even in those studies that have reported an advantage for highly trained musicians, it is not known whether the benefits measured in laboratory tests extend to more ecologically valid situations. This study aimed to establish whether musicians are better than non-musicians at understanding speech in a background of competing speakers or speech-shaped noise under more realistic conditions, involving sounds presented in space via a spherical array of 64 loudspeakers, rather than over headphones, with and without simulated room reverberation. The study also included experiments testing fundamental frequency discrimination limens (F0DLs), interaural time differences limens (ITDLs), and attentive tracking. Sixty-four participants (32 non-musicians and 32 musicians) were tested, with the two groups matched in age, sex, and IQ as assessed with Raven’s Advanced Progressive matrices. There was a significant benefit of musicianship for F0DLs, ITDLs, and attentive tracking. However, speech scores were not significantly different between the two groups. The results suggest no musician advantage for understanding speech in background noise or talkers under a variety of conditions.

## Introduction

Understanding speech in a noisy environment is a crucial skill for much of human communication, but it is one that becomes more challenging with age. Some studies have suggested that musical training is associated with improved speech perception in noise^1–3^, and that the benefit of musical training may protect against some of the deleterious effects of age on speech perception in noise^4^. However, although it is generally accepted that musical training is associated with improved skills relevant for music, such as pitch discrimination^5–9^, pitch interval discrimination^10,11^ and rhythm discrimination^12,13^, its association with speech perception in noise and other challenging conditions remains disputed because of several failures to find such an effect^7,8,14,15^.

One reason for these discrepancies in outcomes might be differences in speech material, the number and types of maskers, and other parameters (such as degree of spatial separation between target and maskers) that have varied across studies. However, some discrepancies exist even between studies that used similar approaches and stimuli. For instance, of the two studies that used a masker consisting of a single talker^14,16^, one used English sentences spoken by a female target and a male masker and found no significant benefit of musicianship^14^, whereas the other found a significant musician advantage in all conditions when using Dutch sentences spoken by the same male talker as both target and masker^16^. These contradictory results cannot be explained by differences in target-masker similarity between the two studies, because the effects of systematic variations in differences in average fundamental frequency (F0) and vocal tract length did not interact with musical training^16^. There is similar disagreement among studies that measured speech perception using the Quick Speech-In-Noise (QuickSIN) test^1,7,12,17^, a non-adaptive test that assesses speech perception using sentences with few contextual cues in a four-talker babble (three females and one male)^18^. Studies by Parbery-Clark *et al*.^1,3^, Slater and Kraus^12^, and Slater *et al*.^17^ found a small but significant musician advantage (<1 dB) in at least one of the conditions they tested, whereas a study by Ruggles *et al*.^7^ found no such effect. Another example highlighting such inconsistency is a study by Deroche *et al*.^19^, which found a significant musician advantage in only two out of four experiments, despite using similar stimuli and many of the same listeners in all four experiments. Inconsistencies across studies using the same or similar speech material suggest that differences in the number of maskers or target-masker similarity cannot explain the differences in outcomes. Instead, such inconsistencies, and the often small differences between groups, suggest that the musician advantage effect, if it exists, is not very robust.

Another possible source of variation is the spatial relationship between the target and maskers. Studies by Swaminathan *et al*.^20^ and Clayton *et al*.^21^ reported a sizeable musician advantage in a condition where the target was presented directly in front of the listener and the two speech maskers were presented at an azimuth of ±15° relative to the target. No such difference was found when the target and the two unprocessed speech maskers were all presented from the front (colocated). Swaminathan *et al*.^20^ argued that the spatially separated condition used in their study reflects a more ecologically valid situation than the typical case, where the target and masker(s) are colocated in space. However, other aspects of their stimuli were not as ecologically valid, such as the matrix-type speech corpus that was used for both target and masker (where the words are selected from a small closed set); the use of non-individualized head-related-transfer functions (HRTFs) to simulate spatial separation, which leads to limited externalisation^22^; and the lack of reverberation, such as would be encountered in real rooms and other enclosures. It is therefore possible that any musician advantage would be different under more ecologically valid conditions that include more natural differences between talkers, more natural spatial cues, and reverberation.

The present study assesses whether there is an association between musical training and speech perception abilities under more natural conditions than have been typically tested. Speech intelligibility was measured in a large anechoic chamber, where sound was presented via a spherical array of 64 loudspeakers. Speech perception was measured in a background of speech from two competing talkers or speech-shaped noise in conditions where the target and maskers were either colocated or spatially separated in azimuth by ±15°. Conditions with and without reverberation were tested. This study also included another speech task with stimuli and conditions similar to the ones used by Swaminathan *et al*.^20^ and Clayton *et al*.^21^ to determine whether it is possible to replicate their findings with a larger number of participants. Furthermore, psychoacoustic tasks, involving the measurement of F0 discrimination limens (F0DLs), interaural time difference limens (ITDLs), and attentive tracking, were included to assess their relation to musical training and to determine whether the results from these tasks could predict performance in the speech tasks.

## Results

### Psychoacoustic experiments

**Figure 1:**
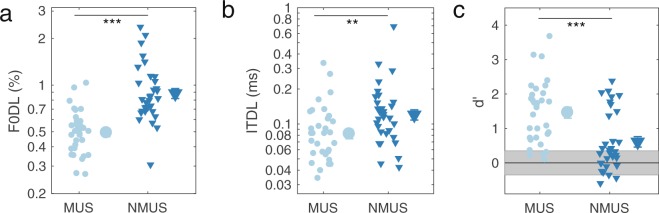
Results from the psychoacoustic experiments. (**a**) Fundamental frequency difference limens (F0DLs), (**b**) Interaural time difference limens (ITDLs), and (**c**) Attentive tracking performance. The grey area in c represent the 95% confidence intervals around chance level. Smaller symbols represent individual data and the larger symbols represent the group means. Error bars represent ±1 standard error of the mean across participants.

Performance on the psychoacoustic tasks by the musician and non-musician groups was compared using two-tailed Welch t-tests. The F0DLs for complex tones with an F0 of 110 Hz, corresponding to the long-term average F0 of the target speech in the open-set speech materials, confirmed that F0 discrimination abilities are significantly better for musicians than for non-musicians (t_58.22_ = 6.21, p < 0.0001, Cohen’s d = 1.55) (Fig. 1a). In addition, ITDLs were significantly better (lower) for the musicians than for the non-musicians (t_61.69_ = 2.71, p < 0.01, Cohen’s d = 0.68), despite large within-group variability and considerable between-group overlap (Fig. 1b). Moreover, the results from the attentive tracking task^23^ showed that the ability to track one sound source in the presence of another varying on three dimensions was significantly better in musicians than in non-musicians (t_61.81_ = 3.83, p = 0.0003, Cohen’s d = 0.96). In fact, the performance of many of the non-musicians was around chance level (*d*′ = 0), leading to something of a bimodal distribution, whereas most musicians performed above chance level (Fig. 1c). Given the non-normal distribution of the non-musicians’ *d*′ values, we also carried out a non-parametric test to test for differences between the two groups (Wilcoxon rank-sum test). The results of this test confirmed a significant effect of group (W = 244.5, p = 0.0003).

### Speech perception tasks

#### Closed-set speech-on-speech task

**Figure 2:**
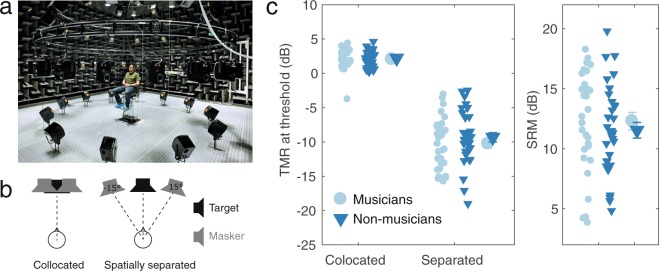
Setup and results for the closed-set speech task. (**a**) The audio-visual immersion lab where the two speech tasks were conducted. Photo courtesy of Torben Nielsen and DTU Elektro. (**b**) Schematic illustration of the spatial conditions used in the two speech tasks. (**c**) Results from the closed-set speech task. Small symbols represent individual results and large symbols represent the group means. Error bars represent ±1 standard error of the mean across participants. The left panel shows the target-to-masker ratio (TMR), which equals the difference in level between the target and one of the two maskers at 50% intelligibility. The right panel shows the spatial release of masking (SRM), which is the difference in threshold TMR between the colocated and spatially separated conditions.

With closed-set target sentences presented from the front (Fig. 2a,b), performance was better (lower target-to-masker ratios, TMRs, at threshold) when the target and maskers were spatially separated than when they were colocated (Fig. 2c). However, overall thresholds, as well as the difference in thresholds between the colocated and the separated maskers (known as the spatial release from masking, SRM), were similar for the musicians and non-musicians. Statistical analysis of the TMRs at threshold, using a mixed-model analysis of variance (ANOVA) with a within-subjects factor of spatial separation and between-subjects factor of group (musicians and non-musicians), confirmed a significant effect of spatial separation (F_1,62_ = 600.34, p < 0.0001, η_G_^2^ = 0.83). However, neither the main effect of listener group (F_1,62_ = 1,29, p = 0.26, η_G_^2^ = 0.011) nor its interaction with spatial separation (F_1,38_ 0.61, p = 0.44, η_G_^2^ = 0.0048) was significant.

There was no correlation (Pearson, two-tailed) between the mean scores across conditions in this speech task and IQ scores measured with Raven’s Advanced Progressive Matrices (r = −0.16, p = 0.22; Supplementary Fig. S1). Furthermore, the correlation between speech scores and the tonal music aptitude scores measured with the Advanced Measures of Musical Audiation (AMMA) test did not reach significance (r = −0.24, p = 0.053; Supplementary Fig. S1) when removing the participant with the highest (worst) speech score, who otherwise drove the correlation (r = −0.28, p = 0.024).

To further investigate the relationship between musical training and speech scores, the data from the musicians were considered alone. The age of onset of musical training was added as a covariate but there was no effect of onset age (F_1,30_ = 0.03, p = 0.85, η_G_^2^ = 0.0005) and no interaction between onset age and spatial separation (F_1,30_ = 0.17, p = 0.69, η_G_^2^ = 0.003), perhaps in part because of our strict selection criteria, meaning that the range of onset ages was small. Similarly, when adding number of years of training as a covariate there was neither an effect of years of training (F_1,30_ = 0.13, p = 0.72, η_G_^2^ = 0.002) nor a significant interaction between years of training and spatial separation (F_1,30_ = 0.57, p = 0.46, η_G_^2^ = 0.01). Finally, an estimate of total hours of practice during their life span, obtained from the Montreal Musical History questionnaire^24^ was added as a covariate, excluding the two musicians who did not answer the relevant questions in the questionnaire. Again, there was no significant effect of hours of practice (F_1,28_ = 1.37, p = 0.25, η_G_^2^ = 0.02) or interaction between hours of practice and spatial separation (F_1,28_ = 0.87, p = 0.36, η_G_^2^ = 0.02).

#### Open-set speech-on-speech and speech-in-noise tasks

**Figure 3:**
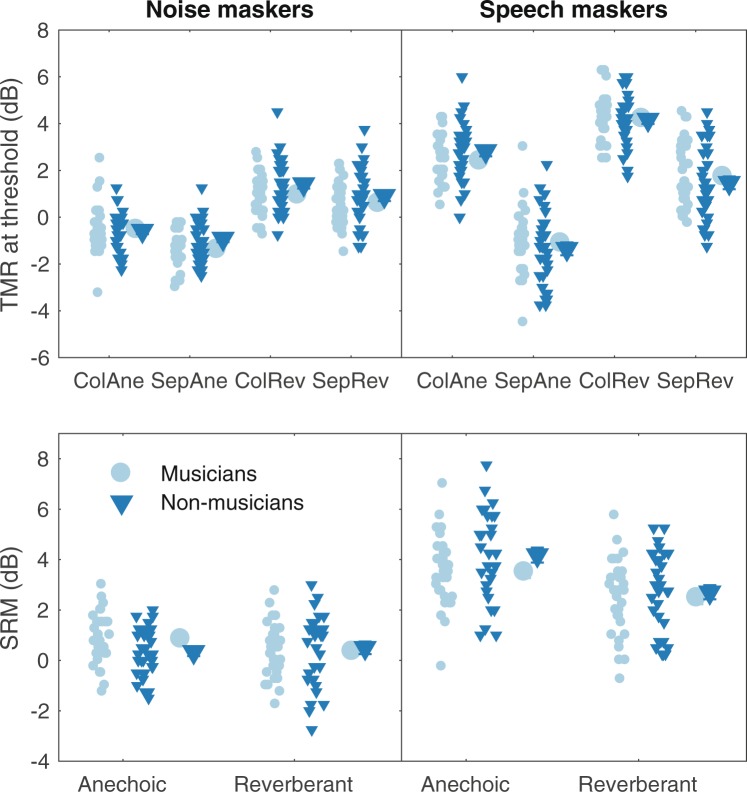
Results from the speech perception task using open-set target sentences. Results from conditions with noise maskers are shown to the left and results with two speech maskers are shown to the right. The upper panels show TMRs at threshold for the conditions: colocated and anechoic (ColAne), separated and anechoic (SepAne), colocated and reverberant (ColRev), and separated and reverberant (SepRev). The lower panels show spatial release of masking (SRM) for the anechoic and reverberant conditions, respectively. The smaller symbols represent individual results and the larger symbols indicate the group means. Error bars represent ±1 standard error of the mean across participants.

The results using open-set target sentences, presented from the front, reflect several expected trends (Fig. 3). First, with both noise maskers (upper left panel) and speech maskers (upper right panel), performance was better (lower TMRs at threshold) when the maskers were separated from the target than when they were colocated, as shown by the positive difference in thresholds between colocated and separated conditions, or SRM (Fig. 3, lower panels). Second, the amount of SRM was greater for speech maskers than for noise maskers (Fig. 3 lower left and right panels). Third, introducing reverberation resulted in somewhat higher thresholds overall. However, none of the data suggest a difference between musicians and non-musicians.

The data were analysed using a mixed-model ANOVA, with TMR at threshold as the dependent variable, reverberation, spatial separation, and masker type as within-subjects factors, and listener group as a between-subjects factor. The analysis confirmed that there were significant effects of reverberation (F_1,62_ = 680.05, p < 0.0001, η_G_^2^ = 0.45), spatial separation (F_1,62_ = 365.99, p < 0.0001, η_G_^2^ = 0.41), and masker type (F_1,62_ = 250.22, p < 0.0001, η_G_^2^ = 0.38). Moreover, the interactions between spatial separation and reverberation (F_1,62_ = 32.09, p < 0.0001, η_G_^2^ = 0.025), reverberation and masker type (F_1,62_ = 4.79, p < 0.032, η_G_^2^ = 0.0075), and spatial separation and masker (F_1,62_ = 213.11, p < 0.0001, η_G_^2^ = 0.27) were all significant. However, there was no effect of listener group (F_1,62_ = 0.65, p = 0.42, η_G_^2^ = 0.0034) and no significant interaction between listener group and reverberation (F_1,62_ = 0.031, p = 0.86, η_G_^2^ < 0.0001), masker type (F_1,62_ = 2.29, p = 0.14, η_G_^2^ = 0.0055), or spatial separation (F_1,62_ = 0.44, p = 0.51, η_G_^2^ < 0.001). Furthermore, there were no significant three-way interactions between listener group, reverberation and separation (F_1,62_ = 0.17, p = 0.69, η_G_^2^ = 0.0001), listener group, reverberation, and masker type (F_1,62_ = 1.48, p = 0.23, η_G_^2^ = 0.002), listener group, spatial separation, and masker masker type (F_1,62_ = 2.91, p = 0.09, η_G_^2^ = 0.005), and no four-way interaction between listener group, reverberation, spatial separation, and masker type (F_1,62_ = 3.12, p = 0.08, η_G_^2^ = 0.004). This result indicates that the two listener groups were similarly affected by reverberation, masker type, and spatial separation and therefore that SRM was also similar for the two groups in this experiment. Thus, the results obtained in this experiment provide no evidence for a musician advantage in understanding speech in noise or speech backgrounds across a wide range of listening conditions.

The mean speech scores, averaged across all conditions within each subject, were not correlated (Pearson, two-tailed) with the IQ scores (r = −0.1, p = 0.43; Supplementary Fig. S2) or the tonal musical aptitude (AMMA) scores (r = −0.19, p = 0.13; Supplementary Fig. S2). As with the closed-set sentences, to further explore the relationship between musical training and speech perception, the data from the musicians were considered alone. When considering the onset age of training as a covariate, there was no effect of onset age or interaction with onset age (Supplementary Table S1). Similarly, when adding the total hours of practice during their life span for the 30 musicians who filled out this part of the survey, there was no main effect of, or interaction with, hours of practice (Supplementary Table S2). Finally, when adding instead the number of years of training as a covariate, there was no main effect of years of training, but there was a significant interaction between years of training and reverberation (F_1,30_ = 7.45, p = 0.01, η_G_^2^ = 0.02; Supplementary Table S3). This relationship was further investigated by correlating the difference between speech scores obtained in the reverberant conditions and scores obtained in the anechoic conditions with their number of years of training for each participant. This analysis revealed a general tendency for the speech scores to be more affected by reverberation with increasing number of years of training (r = 0.45, p = 0.01, two-tailed). However, although the tendency remained the same, this correlation was no longer significant when removing the two participants with the lowest (best) speech scores (r = 0.35, p = 0.06, two-tailed). This trend, suggesting a deleterious effect of musical training on speech perception in a reverberant environment, does not support the idea of a musician advantage in the ability to understand speech in a noisy environment. In addition, given its relatively small effect size, its dependence on extreme data points, and the lack of any correction for multiple comparisons, it seems likely that this correlation is spurious.

## Discussion

The results from this study provide no evidence of a beneficial effect of musical training on the ability to understand speech masked by speech or noise in any of the conditions tested. Thus, the presence of a musician advantage does not seem to depend on the type of speech material, spatial separation, or reverberation. In fact, a power calculation showed that in order to obtain a significant difference between groups with statistical power at the recommended 0.80 level using the effect size estimated from our data, we would need 554 participants for the closed-set matrix test experiment and 1298 participants for the open-set experiment.

**Figure 4:**
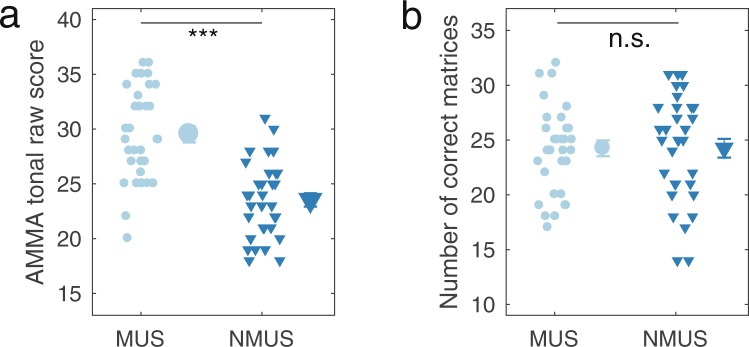
Scores from tests performed as part of the screening procedure. (**a**) Scores from the tonal part of the Advanced Measures of Music Audiation (AMMA) test. (**b**) Scores obtained using Raven’s Advanced Progressive Matrices.

No significant relationship was found between speech scores and our measure of musical aptitude, the tonal AMMA scores, which have previously been shown to relate to anatomical and physiological cortical differences between groups of non-musicians, amateur musicians, and professional musicians^25^. The AMMA scores are not a reliable indicator of the amount of musical training of the individual, as is reflected by the considerable overlap in the AMMA scores between groups, despite the large difference in amount of musical training (Fig. 4). This overlap and the large range of scores are consistent with the finding of a recent study showing high musical aptitude for some non-musicians but not for others^26^. Although that study did not consider the correlation between musical aptitude and speech scores, it did report similar speech scores for a group with high and a group with low musical aptitude scores, consistent with the lack of correlation found in the present study. However, that study did report an enhanced neural encoding of speech signals in non-musicians with high musical aptitude^26^. It may be that neural enhancements do not necessarily correspond to a marked improvement in the ability to understand speech in a noisy environment.

Considering the large musician advantages found by Swaminathan *et al*.^20^ and Clayton *et al*.^21^, it was somewhat surprising that we did not find a significant musician advantage in the spatially separated condition, particularly when using the closed-set speech material. This apparent discrepancy between those previous studies and the present one might be related to factors such as the different speech materials in different languages and, perhaps more importantly, the fact that the stimuli were presented over loudspeakers in our study but were presented via headphones using non-individualized head-related transfer functions (HRTFs) to simulate spatial separation in the previous studies. The use of generic HRTFs might have led to limited externalization, whereas the use of loudspeakers might have led to differences in the exact position of the participants’ head, relative to the sound sources. However, such deviations in head positions would have been small since the participants were asked to sit straight and still while facing the loudspeaker in front of them, with the position monitored throughout the experiment via a video camera inside the testing room. A close comparison of threshold TMRs across studies suggests that thresholds are similar for the spatially separated condition for the non-musicians, but differ markedly for the musicians. In our study, the musician’s thresholds were highly variable and were similar to those of the non-musicians. In contrast, in the Swaminathan *et al*. study^20^, the musicians’ thresholds were much less variable and were at least as low (good) as those of the best-performing non-musicians. However, despite using exactly the same stimuli and even some of the same participants, the benefit of musicianship was much less pronounced in the study of Clayton *et al*.^21^. That study did find an overall musician advantage but the musicians’ thresholds were more variable and more similar to those of the non-musicians. The high variance between thresholds in the spatially separated condition and the differing results across studies highlights the need for large sample sizes when testing hypotheses related to musical training. The numbers of subjects tested by Swaminathan *et al*. (N = 24) and Clayton *et al*. (N = 34) were considerably smaller than the number tested here (N = 64).

The psychoacoustic experiments included in this study provide evidence of a musician advantage in auditory tasks, specifically F0 discrimination, ITD discrimination, and attentive tracking. Many previous studies have shown enhanced F0DLs; however, to our knowledge, no previous studies have compared ITDLs and attentive tracking in musicians and non-musicians. It is especially interesting that the benefit seen in the attentive tracking task is not reflected in the speech data. However, one difference between the psychoacoustic experiments and the speech tasks is that none of the participants had previously received explicit training in the psychoacoustic tasks. Also, it may be that musicians have experience in making fine-grained auditory discrimination judgments, thereby providing them with a benefit in the two discrimination tasks. Evidence in favor of this hypothesis comes from a study by Micheyl *et al*.^5^, which showed a similar musician advantage for F0 discrimination, but also showed that 6–8 hours of training was sufficient for non-musicians to achieve the same high levels of performance on the task as professional musicians. In contrast, all participants have received extensive training in understanding speech in noisy situations in their everyday lives, perhaps leaving little additional benefit to be gained from musical training. The results from this and previous studies indicate that any musician advantage in understanding speech in noise or other background sounds is not robust and is not readily replicated. Considering this outcome and the intense and sustained training of the musicians participating in these studies, it seems unlikely that musical training will be effective as a clinical tool for improving the ability to understand speech in noisy situations.

## Methods

### Participants

64 participants (32 musicians and 32 non-musicians) were tested. The musicians were required to have started musical training at or before the age of 7 years, to have received musical training for at least 10 years, and to still play or sing at least 5 hours per week. More information about the musicians can be found in Table 1. The non-musicians were required to not have played an instrument or sung for more than two years, and to not have actively played or sung within the last 7 years. All participants were native Danish speakers and had audiometric thresholds at octave frequencies between 250 and 8000 Hz no greater than 20 dB HL. As shown in Table 2, the groups were matched in gender, age, and IQ (Raven’s Advanced Progressive Matrices). The latter was measured as the number of matrices correctly solved within 30 minutes. The musical aptitude of the participants was also tested with the Advanced Measures of Music Audiation (AMMA) test^27^. In each trial, the participants heard a musical phrase twice and were asked to indicate whether the phrase changed. If it changed, the participants had to indicate whether the change was rhythmic or tonal. The test provides a tonal and a rhythmic score. The results from the AMMA and IQ tests are shown in Fig. 4.

**Table 1:** Overview over the musical experience of the musicians tested in this study.

#	Age of onset (years)	Years of training	Accumulated hours of practice	Primary instrument
1	6	14	14678	Accordion
2	5	18	n/a	Violin
3	6	16	10088	Trombone
4	6	10	3216	Piano
5	7	20	3216	Trumpet
6	6	13	3216	Piano
7	5	13	5084	Double bass
8	7	11	n/a	Voice
9	7	10	12324	Electric bass
10	7	19	17454	Violin
11	6	17	17360	Double bass
12	7	16	15912	Trumpet
13	7	19	6568	Drums
14	6	16	5616	Voice
15	4	16	8920	Viola
16	6	20	14476	Piano (choir director)
17	6	15	832	Oboe
18	7	16	2368	Voice
19	6	14	7848	Drums
20	6	22	14664	Guitar
21	6	12	4718	Guitar
22	7	12	738	Voice
23	6	14	14112	piano
24	6	12	8370	Electric bass
25	7	15	4509	piano
26	7	19	1529	Trombone
27	6	13	1394	Oboe
28	7	12	9472	Guitar
29	4	13	1498	Trumpet
30	4	15	8631	Piano
31	7	18	1025	Piano
32	6	18	4992	Piano

**Table 2:** Demographic information. Table presents the group averages.

	Musicians (N = 32)	Non-musicians (N = 32)	p-value
Age (years)	22.84 (3.48)	22.94, (2.2)	0.9
Sex	16 females, 16 males	17 females, 15 males	0.80
IQ (Number of correctly answered matrices)	24.25 (4.1)	24.31 (4.84)	0.96
Musical aptitude (Tonal AMMA score)	29.53 (4.25)	23.53 (3.46)	<0.0001

The standard deviations are shown in the parentheses. The table also show p-values for comparison of the two groups. Independent-samples t-tests were used to compare age, IQ, and AMMA scores and a χ2 test was used to compare distribution of gender distribution in the two groups. IQ was measured using Raven’s Advanced Progressive Matrices and musical aptitude was assessed using the tonal score obtained in the Advanced Measures of Musical audiation (AMMA) test.

All subjects provided informed consent prior to their participation in the experiments. The experimental protocols were approved by the Scientific Ethical Committees of the Capital Region of Denmark (H-16036391) and were carried out in accordance with the corresponding guidelines and relevant regulations on the use of human subjects.

### General methods

The order of the stimuli in the attentive tracking and the order of the conditions in the two speech experiments were randomized across participants in each group but was always the same for one musician and one non-musician.

All experiments other than the speech tasks were conducted in a double-walled acoustically shielded booth. The stimuli were generated in MATLAB (The Mathworks, Natick, MA, USA) at a sampling rate of 48000 Hz and presented via a Fireface UCX sound card (RME, Haimhausen Germany) and Sennheiser HD 650 headphones (Sennheiser, Wedemark, Germany).

The speech tasks were conducted in a large anechoic chamber (7 m*8 m*6 m) using a virtual sound environment (VSE), with a spherical array of 64 loudspeakers^28^ (Fig. 2a) to render the stimuli in a more realistic manner. Results are reported as the target-to-masker-ratio (TMR) at which 50% of the words are reported correctly by the participants. Spatial release of masking (SRM) was calculated as the difference between the thresholds obtained in the colocated and the spatially separated conditions.

Informed consent for publication of identifying images in an online open-access publication was obtained.

### F0 discrimination limens (F0DLs) and Interaural time difference limens (ITDLs)

Both the F0DL and ITDL experiments used a 3-down 1-up, 2-interval 2-alternative forced-choice procedure similar to the one previously used by Madsen *et al*.^8^. Each interval contained four consecutive harmonic complex 200-ms tones that were each gated on and off with 20-ms raised-cosine ramps. All tones were shaped spectrally to have the same long-term spectral envelope as the target in the open-set speech task and were presented at 55 dB SPL in each ear. For each run, the threshold was calculated as the geometric mean of the values at the last six reversal points. The final thresholds for each participant were calculated as the geometric mean across the last three out of four runs. All statistics were performed on the log-transformed thresholds.

For the F0DL experiment, the participants were asked to indicate which interval contained the changes in pitch. All tones were presented diotically. The four tones in the reference interval all had an F0 of 110 Hz to match the average F0 of the target speech. In the target interval, the F0 of the first and the third tone was higher and the F0 of the second and fourth tone was lower than that of the reference tones. The F0 difference between the high and the low tones was varied adaptively on a logarithmic scale and the two F0s were geometrically centered on 110 Hz.

For the ITD experiment, the participants were instructed to indicate in which of the intervals the tones were perceived to move within the head. Here, all tones had an F0 of 110 Hz. The four tones in the reference interval were presented diotically (ITD = 0). In the target interval, an ITD was introduced in the odd tones, with the left side leading, and the opposite ITD was introduced in the even tones, with the right side leading, so that the first and third tone were perceived to the left of the midline and the second and fourth tone were perceived to the right of the midline, leading to the perception of motion between the alternating tones. The ITD was varied adaptively on a logarithmic scale.

### Attentive tracking

Attentive tracking was tested using a paradigm introduced by Woods and McDermott^23^ that tests the ability of participants to follow one of two simultaneous synthetic voice trajectories. In each trial, the mixture of voices was preceded by the first 500 ms of one of the voices, to cue the participants to attend to that voice. The mixture was followed by the last 500 ms of one of the voices (the probe) and the participants were asked to indicate whether this was the end of the cued voice or not (yes or no). The stimuli had a duration of 2 s and varied continuously in F0 and each of the first two formants (F1 and F2). The voices crossed each other in all feature dimensions (F0, F1, and F2) at least once but were always separated by at least 6.5 semitones (Euclidean distance in the three-dimensional feature space). One hundred fixed voice pairs were used and each was presented twice to each participant, once with the correct probe and once with the incorrect probe. The experiment was divided into five blocks of 40 runs and in each block half of the pairs were presented with the correct probe. The order of correct vs incorrect probe was randomized. Furthermore, the order of the voice pairs was randomized such that each voice pair was presented once in the first half and once in the last half of the experiment. Prior to the experiment, the participant heard a few example stimuli. To avoid participants basing their judgements solely on the similarity between the cue and probe, voice pairs were selected for which the average distance in feature space between the cue and the two probes were the same (8.05 semitones). The voices were generated by Klatt synthesis^29^ with parameters similar to the ones used by Woods and McDermott^23^. Thus, the trajectories of each feature were generated from Gaussian noise, filtered between 0.05 and 0.6 Hz, and the features of F0, F1, and F2 spanned ranges of 100–300 Hz, 300–700 Hz, and 800–2200 Hz, respectively. Feature means and SDs (semitones from the mean) were: F0: μ = 180.38 Hz, SD = 4.2 semitones; F1: μ = 466.5 Hz, SD = 4.2 semitones; F2: μ = 1356.6 Hz, SD = 3.9 semitones. The *d*′ values were calculated using the log-linear rule to avoid undefined extremes^30,31^.

### Closed-set speech-on-speech task

Both target and maskers were speech from multi-talker recordings of the Dantale II speech corpus^32,33^. Only recordings from three out of five speakers for whom the average root-mean-square levels were most similar to each other were used (talker 1,4, and 5). Each sentence consisted of five words of the structure “name, verb, numeral, adjective, noun”. The name was used as a call-sign and the participants were asked to identify the remaining four words by selecting the appropriate choices on a touchpad. The call-sign (name) was fixed throughout each TMR measurement but was varied across measurements while the target and masker talkers were varied on each trial. The masker sentences never contained the same words as the target. Scoring was done on a word basis and the target-to-masker ratio (TMR) was adapted to track the point at which 50% of the words were reported correctly^34^. The level of each masker was kept constant at 55 dB SPL and the target level was varied adaptively. Each of the two conditions (colocated and ±15° spatial separation) was tested twice and the order was randomized across participants. Training consisted of one TMR-measurement for each of the two conditions.

### Speech in ecologically valid situations

In this experiment, the target was always presented directly in front of the listeners and the two maskers were either colocated or spatially separated from the target along the azimuthal (horizontal) plane by ±15°. The two spatial conditions were tested in both an anechoic and a reverberant condition. In the latter, the reverberation in a standard listening room^35^ was simulated using ODEON software (version 13.04; Odeon A/S, 10 Denmark) and reproduced in the VSE using nearest loudspeaker playback^36^. Each condition was tested twice for each listener.

The TMRs were measured using CLUE sentences^37^ for the target. These are short HINT^38^-like sentences with some context.The masker was either a Gaussian noise, spectrally shaped to have the same long-term spectrum as the target speech, or a two-talker masker, also with the same long-term spectrum as the target speech. The speech maskers were made from conversations recorded by Sørensen *et al*.^39^ after removing all gaps exceeding 100 ms, non-Danish words, loud exclamations, and other sounds such as laughter. All speakers were male. The target sentences had an average F0 of 110 Hz while the average F0 of the two maskers were 143 and 146, respectively. In order to reduce the F0 difference between the target and maskers to 2 semitones, the maskers were manipulated with PRAAT^40^. The CLUE sentences had a duration of between 1.23 and 1.86 s. The maskers started 500 ms before and ended at least 100 ms after the target and were gated with 50 ms raised-cosine onset and offset ramps. During the experiment, the experimenter scored the test outside the anechoic chamber. The participants were instructed to repeat as much as they could of the target sentence after each trial. They were guided towards the target voice by the presentation of one CLUE sentence (always the same) in quiet immediately before each trial. The masker level was kept constant at 55 dB SPL and the target level was varied adaptively. Each sentence list contained 10 sentences and the level of the target always started at 50 dB SPL and was increased by 2 dB until the entire sentence was repeated correctly. In the following trials, the target level was varied adaptively in steps of 2 dB using a 1-up 1-down procedure resulting in the 50% correct threshold. The sentences were scored according to the rules suggested by Nielsen and Dau^37^, allowing for change in verb tense, change in article, and change between singular and plural nouns. Additional words and the specific alternatives of de/vi (they/we), hun/han (he/she), and min/din (my/your) were accepted. The participants were trained with two lists that together covered the range of tested conditions, with one training list presented in an anechoic, colocated condition with a noise masker and the other training list presented in a reverberant condition with spatially separated speech maskers.

## Supplementary information

Supplementary Information

## Data Availability

The datasets generated and analyzed during the current study, along with the analysis code, are available from the corresponding author upon reasonable request.
