# Supplementary material for: Speech perception is similar for musicians and non-musicians across a wide range of conditions
Source: Sci Rep. 2019 Jul 18;9:10404. doi: 10.1038/s41598-019-46728-1 (PMC6639310; doi:10.1038/s41598-019-46728-1)
Supplement: Supplementary file 1 — Supplementary Information [file 41598_2019_46728_MOESM1_ESM.pdf]

Speech perception is similar for musicians and non-musicians across wide range of conditions

Sara M. K. Madsen, Marton Marschall, Torsten Dau and Andrew J. Oxenham

Effect	F	p-value	η_G^2
Onset age	1.88	0.18	0.02
Onset age x reverberation	1.80	0.19	0.005
Onset age x spatial separation	0.06	0.81	0.0002
Onset age x Masker	0.91	0.35	0.005
Onset age x reverberation x spatial separation	0	0.94	<0.0001
Onset age x reverberation x masker	0.63	0.44	0.001
Onset age x separation x masker	1.14	0.29	0.005
Onset age x reverberation x spatial separation x masker	1.01	0.32	0.002

Supplementary Table S1:Results obtained when adding onset age as a covariate.

Effect	F	p-value	η_G^2
Hours of practice	2.68	0.11	0.03
Hours of practice x reverberation	0.46	0.50	0.001
Hours of practice x spatial separation	0.82	0.37	0.003
Hours of practice x Masker	0.00	0.97	<0.0001
Hours of practice x reverberation x spatial separation	3.23	0.08	0.006
Hours of practice x reverberation x masker	0.06	0.80	0.0001
Hours of practice x separation x masker	0.12	0.73	0.0006
Hours of practice x reverberation x spatial separation x masker	0.59	0.45	0.001

Supplementary Table S2: Results obtained when adding accumulated hours of practice as a covariate

Effect	F	p-value	η_G^2
Years of training	0.06	0.81	0.0006
Years of training x reverberation	7.45	0.01	0.02
Years of training x spatial separation	1.41	0.24	0.05
Years of training x Masker	0.06	0.80	0.0004
Years of training x reverberation x spatial separation	0.32	0.58	0.0006
Years of training x reverberation x masker	1.44	0.24	0.003
Years of training x separation x masker	0.01	0.93	<0.0001
Years of training x reverberation x spatial separation x masker	3.67	0.06	0.007

Supplementary Table S3: Statistical values obtained when adding years of training as a covariate

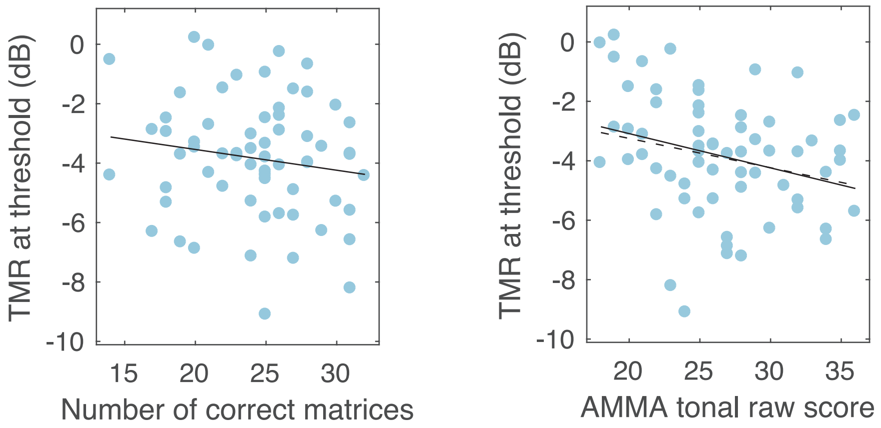

Supplementary Figure S1: Correlation between the average closed-set speech scores and the number of correct Raven's matrices (r = -0.16, p = 0.22; left) and the tonal AMMA score (right). Regression lines are shown for all participants (solid line; r = -0.28, p = 0.024) and for 58 participants (dashed line; r = -0.24, p = 0.053).

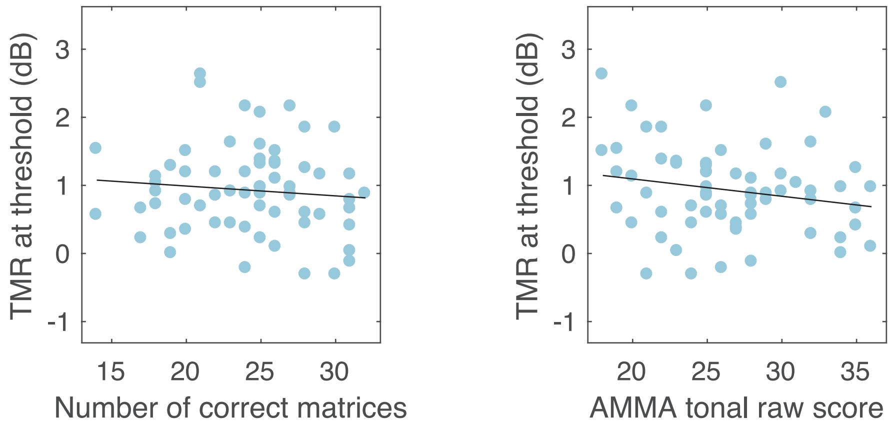

Supplementary Figure S2: Correlation between the average open-set speech scores and the number of correct Raven's matrices (r = -0.1, p = 0.43; left) and the tonal AMMA score (r = -0.19, p = 0.13; right).
